# Features and Educational Content Related to Milk Production in Breastfeeding Apps: Content Analysis Informed by Social Cognitive Theory

**DOI:** 10.2196/12364

**Published:** 2019-05-01

**Authors:** Suhail Sidhu, Kaoer Ma, Anna Sadovnikova

**Affiliations:** 1 LiquidGoldConcept, Inc Ypsilanti, MI United States; 2 School of Medicine University of California, Davis Sacramento, CA United States

**Keywords:** milk production, milk supply, human lactation, breastfeeding, breastfeeding app, smartphone app, Social Cognitive Theory, breastfeeding self-efficacy

## Abstract

**Background:**

Low milk production is one of the main reasons for premature breastfeeding cessation. Smartphone apps have the potential to assist mothers with promoting, interpreting, tracking, or learning about milk production. It is not known whether breastfeeding apps contain high-quality, engaging, and diverse content and features that could be used by mothers to increase their breastfeeding self-efficacy and answer their questions about milk production.

**Objective:**

The overarching objective of this study was to identify and evaluate features and content within breastfeeding apps that could be used by mothers to increase breastfeeding self-efficacy and answer their questions about milk production. The secondary objectives were to quantify the diversity of representation of breastfeeding experiences within breastfeeding apps and to define the type of organization that is most likely to create free apps and/or apps with high-quality, engaging, and diverse features and content related to milk production.

**Methods:**

Breastfeeding apps were identified in the Apple App Store. All features that assist mothers with tracking, promoting, or interpreting milk production in the first 0-6 months postpartum were noted. Every screen containing educational information about milk production was identified and saved for review. Images of mothers and caretakers within the selected screenshots were assessed. Three scoresheets informed by Social Cognitive Theory were created to evaluate all identified features, educational content, and images representing the breastfeeding experience.

**Results:**

Forty-one breastfeeding apps were reviewed. Only seven apps contained both features and educational content related to milk production. Thirteen apps that contained educational content related to milk production received a mean combined content and cultural diversity score of 15.3 of 78. Of the 48 photos reviewed in screenshots that contained educational content on milk production, 87.5% (n=42) were of white women and their infants. For-profit companies and large organizations were most likely to create free apps and apps that received high scores on the combined content and diversity or features scoresheet, respectively.

**Conclusions:**

Features and educational content related to milk production and breastfeeding imagery within breastfeeding apps were evaluated using three novel scoresheets informed by Social Cognitive Theory. Few apps contained both features that promote breastfeeding self-efficacy and high-quality, engaging, educational content with images of diverse caretakers. Thus, it is likely that parents, especially those from minority or low-income groups, have limited options when selecting a breastfeeding app. App developers could use the scoresheets and findings in this review to develop breastfeeding apps that assist mothers with interpreting, tracking, or learning about milk production through high-quality and engaging features, content, and imagery.

## Introduction

Breastfeeding significantly reduces maternal and infant morbidity and mortality [1]. Despite the fact that the World Health Organization and the American Academy of Pediatrics recommend exclusive breastfeeding for 6 months, only 24.9% of mothers in the United States reach the 6-month goal [2-4]. Racial and socioeconomic disparities are reflected in breastfeeding rates: Black and low-income women are less likely to initiate breastfeeding in the hospital and breastfeed exclusively for 6 months [5]. The majority of women stop breastfeeding due to perceived or real low milk production [6,7].

A health behavioral intervention that focuses on maternal self-efficacy is an effective strategy for increasing breastfeeding rates [8]. A mother’s breastfeeding self-efficacy could improve if she had the ability to track, interpret, and learn about her milk production [9]. Women are turning to their smartphones [10] to seek out “emotional, informational, technical, and consultative-type breastfeeding support,” information about milk production [11], and visual representations of breastfeeding skills [12]. It is not known whether features and content related to milk production within breastfeeding apps are engaging, diverse, and of high quality or lead to increased breastfeeding self-efficacy [13].

The overarching objective of this study was to identify breastfeeding smartphone apps that contain high-quality, engaging, and diverse content and features that mothers could use to increase their breastfeeding self-efficacy and answer their questions about milk production. The secondary objectives were to quantify the diversity of representation of breastfeeding experiences within breastfeeding apps and to define the type of organization that is most likely to create a free app and apps with high-quality, engaging, and diverse features and content related to milk production.

## Methods

### Identification of Breastfeeding Apps and General Characteristics of App Developers

Between August and October 2017, breastfeeding apps were identified in the Apple App Store using the search term “breastfeeding.” Apps were downloaded onto an iPhone and explored by author SS for 10 minutes to ensure that the app fit the inclusion criteria (Table 1). The Google Play Store was used to confirm that an app could also be downloaded on Android devices; however, availability on both platforms was not a prerequisite for inclusion because the authors only had access to iPhones. To ensure comparability with previous reviews of breastfeeding apps, general information about the app and the app developer was collected from the App Store, by emailing the creators, browsing through the app developer’s page on LinkedIn, and using third-party sites like Bizapedia (Multimedia Appendix 1).

### Selection of Features and Content Related to Milk Production

SS reviewed every interface of each app on three separate occasions (average of 60 minutes per app) to ensure that all possible features were included for review (Multimedia Appendix 2). A feature was defined as an opportunity for user interaction with the app (eg, a button). After identifying all the features available in each app, SS documented features that could assist mothers with promoting, tracking, or interpreting milk production in the first 0-6 months postpartum. The principal investigator, a board-certified lactation consultant and a physician-scientist trainee studying milk production regulation, trained SS to identify features that are relevant to milk production promotion, tracking, and interpretation. A literature search in Medline was conducted to confirm that the selected features were relevant to milk production.

The authors developed a scoresheet with eight milk production content categories using two textbooks on breastfeeding [16,17] as guides (Multimedia Appendix 3). The eight content categories are listed below: (1) Hospital practices that promote breastfeeding initiation; (2) Reasons for a delay in lactogenesis II; (3) Normal milk production timeline, volume, and measurement; (4) Supply and demand physiology; (5) Maternal or infant nutritional requirements; (6) Breastfeeding techniques that support or interfere with milk production; (7) Maternal or infant biological, physiological, or behavioral causes of perceived or real low-milk production between 0 and 6 months postpartum; and (8) A description of foods, medications, or supplements that have the potential to increase milk production. AS provided training to SS on how to identify relevant content within each of the eight categories. AS and SS independently took screenshots within each app to capture educational content related to milk production, reviewed the compiled screenshots, scored all the content, and resolved discrepancies by referring to two textbooks on breastfeeding [16,17].

### Development of Scoresheets to Score Features and Content Related to Milk Production and Images of Breastfeeding Experiences

Social Cognitive Theory is the most common health behavior theory used in self-efficacy instruments that increase breastfeeding rates [8]. The three core components of Social Cognitive Theory that relate to improved breastfeeding self-efficacy are *motivation, observation,* and *repetition.* For a new mother to learn the skill of breastfeeding, she must be intrinsically *motivated* to learn, *observe* ideal examples of the behavior by mothers like her, and receive positive reinforcement from credible sources to *repeatedly* perform the said behavior [8,18,19]. The authors applied these three principles in the development of the scoring system used to evaluate features and content related to milk production and images of the breastfeeding experience within selected breastfeeding apps.

**Table 1:** Inclusion criteria and rationale for selection of breastfeeding apps.

Criterion	Rationale
App appeared as a result on the App Store using the search term “breastfeeding”	“Breastfeeding” yielded >100 results, depending on the month of search. “Lactation” yielded only ~50 results, depending on the month of search.
App focused on breastfeeding education or tracking an aspect of the breastfeeding experience	Breastfeeding experience included nursing and pumping.
App had a summary rating on the App Store	The summary rating on the App Store gives a measurement of whether the app is used by the general public. The App Store ranks apps using an algorithm that considers a multitude of factors related to user engagement including the number of downloads, ratings, and updates; quality of ratings; user retention rate; and conversation rate. The App Store provides a special designation to apps ranked in the top 200 of their assigned categories. Top 200 apps are more likely to have higher user engagement in terms of the aforementioned metrics and serve as an objective measure of an app’s popularity by consumers [14,15].
App was available in English	Authors did not have funds to pay for translation.
App was available for download and use in the United States	Authors reside in the United States.

A 5-point scoresheet was developed to score features (Table 2) that assist mothers with promoting, tracking, or interpreting milk production. Features were included in the scoresheet only if they were relevant to tracking or interpreting milk production between 0 and 6 months postpartum; therefore, all the features included in the scoresheet received one point at baseline. Features that needed to be used frequently (*repetition*) and were interactive (*motivation*) received the highest possible features score. The assignment of points for each feature was determined independently by two raters (AS and SS).

A 72-point scoresheet was developed to evaluate educational content across the eight milk production categories (Table 3 and Multimedia Appendix 3). Three criteria were used to score the educational content within each of the eight categories: scope, quality, and engagement. The selected screenshots did not need to contain all the information from a category to receive full credit within that category. For example, within category 1 (Hospital Practices), selected screenshots would only need to contain a mention of the importance of skin-to-skin contact in relation to breastfeeding initiation to receive a point within the scope of content for that category. The quality of the content was determined by assessing in-text citations and referenced literature within the selected screenshots. AS downloaded and reviewed all referenced literature to ensure it was relevant to the content. Photos and videos are more likely than text to increase breastfeeding self-efficacy because they are *motivational* and are more likely to foster *repetition* and, thus, mastery of a task [18-21]. Therefore, if a screenshot contained educational images pertaining to the content within that category, two additional points were awarded. Videos were awarded three additional points because they are more likely to result in mastery of tasks relative to photos [22]. Two authors independently (SS and AS) assessed each screenshot with information about milk production and awarded points using the content scoresheet described in Table 3.

**Table 2:** Feature scoresheet informed by Social Cognitive Theory.

Measure		Points possible
**Repetition (frequency)**		
	≥1/week	2
	≥1/month but less than ≥1/week	1
	<2/month	0
**Motivation (interactivity)**		
	User engagement throughout activity	2
	User engagement at the beginning or end of activity	1
	Minimal user engagement	0
Milk production related		1
Maximum points/feature		5

**Table 3:** Content scoresheet informed by Social Cognitive Theory.

Content	Scope of content^a^	Quality of content^b^	Engagement with content	
			Videos^c^	Images^d^
Hospital Practices	0/1	0/1/2/3	0/3	0/2
Delay in Lactogenesis II	0/1	0/1/2/3	0/3	0/2
Normal milk production timeline or volume	0/1	0/1/2/3	0/3	0/2
Supply and Demand Physiology	0/1	0/1/2/3	0/3	0/2
Nutritional Requirements	0/1	0/1/2/3	0/3	0/2
Breastfeeding Technique	0/1	0/1/2/3	0/3	0/2
Biology or behavior	0/1	0/1/2/3	0/3	0/2
Foods or medications or supplements	0/1	0/1/2/3	0/3	0/2
Maximum total points possible within each evaluation criterion	8	24	24	16

^a^Is this content presented? (0=No relevant content, 1=Relevant content present).

^b^Are in-text, relevant citations included? (3 points/category) OR Is there a list of relevant references at bottom of screen or within the app? (2 points/category) OR Is the author is an “expert” (eg. MD, IBCLC, or PhD)? (1 point/category) OR There is no way to evaluate the quality of the content? (0 points/category).

^c^Are there videos that assist the user in understanding the educational content? (0=No relevant videos, 3=Videos that assist the user in understanding the educational content are present).

^d^Are there images that assist the user in understanding the educational content? (0=No relevant images, 2=Images that assist the user in understanding the educational content are present).

**Table 4:** Diversity scoresheet informed by Social Cognitive Theory.

Diversity of representation of the breastfeeding experience	Additional points/app
≥7 photos (3 points) OR 4-6 photos (2 points) OR 1-3 photos (1 point) of the breastfeeding dyad or caretakers in all the identified screenshots	3/2/1
*Of all of the identified photos*: ≥3 photos are of nonwhite or nontraditional caretakers^a^ (3 points) OR ≥2 photos are of nonwhite or nontraditional caretakers (2 points) OR 1 photo is of a nonwhite or nontraditional caretaker (1 point)	3/2/1
Maximum points possible/app	6

^a^For the purposes of this evaluation, a nontraditional caretaker was defined by the authors as an individual who is not the mother (eg, grandmother, father, same sex partner, nanny, etc).

A scoresheet (Table 4) was developed to calculate the total additional points that an app could receive for including diverse representations of the breastfeeding experience within the selected screenshots containing educational content about milk production. If the selected screenshots contained images of breastfeeding dyads and nontraditional caretakers, additional points were awarded because the *observation* of successful breastfeeding experiences can increase breastfeeding self-efficacy [8,18-20,23-25]. To calculate the final score for educational content, we combined the scores from the content and diversity scoresheets (72+6=78 possible points), as the images selected for review of “diversity” were identified solely within screenshots with content related to milk production. Thus, the measure of diversity is within those screenshots and is not generalizable to the entire app.

### Statistical Analysis

All data were analyzed using descriptive statistics. To test whether there was a difference between the mean features or combined content and diversity scores, apps were stratified by their rank (ranked vs unranked), platform availability (iOS exclusive vs available on iOS and Android), category (medical apps vs all others including education, productivity, lifestyle, and health and fitness), and price (free vs not free). The organizations that created the apps were also stratified by the size of their business (large organizations with >10 employees vs small organizations) and registration status (for-profit organizations vs all others including governmental organizations, nonprofits, and individuals). The Wilcoxon rank sum test was used to test the difference between two groups. The Spearman correlation was performed to test the relationship between organization type and size; app characteristics; and the features, content, and combined diversity and content scores. Interrater reliability for the combined content and diversity score was calculated as percent agreement between two raters—a breastfeeding expert (AS) and a novice (SS). Significance was set at an alpha of 5%. Data are presented as mean (SEM).

## Results

### Selection of Breastfeeding Apps

Between August and October 2017, 105 apps were identified on the App Store by using the search team “breastfeeding” (Multimedia Appendix 4). Thirty-four apps were not downloaded because they did not have enough ratings to generate a summary rating (n=32) or because they were not available in the United States (n=2). SS downloaded and performed an initial screening of the remaining 71 apps by interacting with each app from an end user’s perspective to confirm that the inclusion criteria were satisfied (Table 1). Eighteen apps were excluded from further assessment because they were not available in English (n=2); were free trials that only allowed the user to use the app for a certain amount of time or number of uses or restricted certain features (n=9); or had technical errors that interfered with the app’s functionality, rendering it impossible to use (n=7). Between November 2017 and August 2018, SS reviewed 53 apps that fit the inclusion criteria on three separate occasions to identify features and content relevant to milk production. In 2018, six apps were excluded from the review because they no longer existed (n=4) or were no longer available in the United States (n=2). Of the remaining 47 apps, six apps were removed from the dataset because they did not have educational content related to milk production (Multimedia Appendix 5). Thus, the final dataset contained 41 breastfeeding apps (N=41).

### General Characteristics of Breastfeeding Apps

Of the 41 breastfeeding apps (Multimedia Appendices 6 and 7) in the final dataset, 85.4% (n=35) had features that mothers could use to track or interpret milk production and 31.7% (n=13) had educational content related to milk production. Of the 13 apps with educational content related to milk production, only six apps contained features that could be used to track or interpret milk production. Twenty-nine apps (70.7%) contained features related to milk production, but no educational content related to milk production. Most of the apps (n=30, 73.2%) were free to download and use. Approximately half of the apps reviewed (n=21, 51.2%) were only available on the iOS platform. The majority of the apps (n=28, 68.3%) were created by for-profit organizations. Over half of the apps (n=25, 60.9%) were created by organizations with fewer than 10 employees. The vast majority of the apps in our final dataset (n=30, 73.2%) were not ranked in the top 200 in the App Store in their respective category.

### Features That Assist Mothers With Promoting, Tracking, or Interpreting Milk Production

Eighteen unique features related to promoting, tracking, or interpreting milk production were identified within 35 breastfeeding apps (Table 5). All 35 apps had a breastfeeding timer, 31 apps had a bottle-feeding timer, and 30 apps had a diaper-change tracker. The least common features were trackers for allergy (n=2), teeth (n=3), and baby’ sounds (n=3). A baby sound recorder and photos of the baby received the highest features score possible of 5. The teeth-tracking feature received the lowest features score because, while teeth can interfere with breastfeeding, it is unlikely that many teeth will appear before the newborn is 6 months old; therefore, the tracking teeth feature would not be used frequently. Moreover, entering the date that a tooth first appeared on would not be as engaging as other activities related to tracking, interpreting, or promoting milk production, such as taking or looking at a photo of a baby.

### Characteristics of Apps Containing Features That Assist Mothers With Promoting, Tracking or Interpreting Milk Production

**Figure 1:**
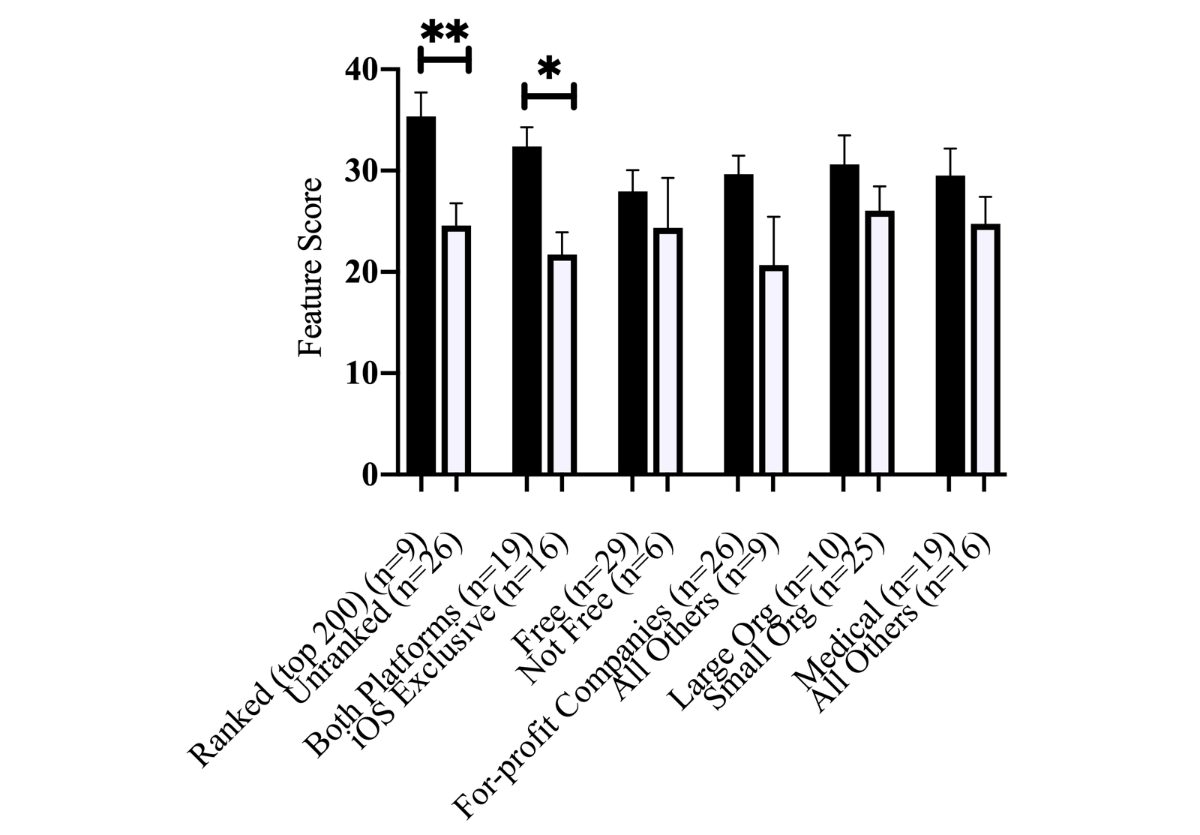
Differences in mean feature scores, stratified by type of app, category (eg, medical or health and fitness), and organization. iOS: iPhone operating system; Org: organization. Asterisk indicates *P*=.04; double asterisk indicates *P*=.0096.

A total of 35 apps contained features that assist mothers with promoting, tracking, or interpreting milk production; these apps received an average features score of 27.3 (SD 11.3; range 4-47 of 51 possible points) when evaluated with the features scoresheet described in Table 5. Among apps that contained features related to milk production, those ranked in the top 200 in their respective category in the App Store (Figure 1) scored significantly higher (*P*=.0096) than apps that were unranked. Apps developed for both iOS and Android (Figure 1) received a significantly higher features score (*P*=.04) than apps developed just for iOS. There was no difference (*P*=.55) between the mean features scores of apps that were free and not free or the mean scores of apps (*P*=.55) created by small versus large organizations (Figure 1). Apps created by large organizations (>10 employees) were more likely to be on both iOS and Android platforms (*r*=0.69, *P*<.001). For-profit companies were more likely to create apps on both iOS and Android platforms (*r*=0.41, *P*=.02). Apps that had to be purchased (“not free apps”) were more likely to be ranked in the top 200 in their respective category in the App Store (*r*=0.43, *P*=.01). There was no correlation between the price of the app and the size of the organization, the organization type, or platform. Additionally, we did not find a significant positive or negative correlation between the size of the organization and its status as a for-profit organization.

### Content Related to Milk Production and Diverse Representations of Breastfeeding Within Breastfeeding Apps

**Figure 2:**
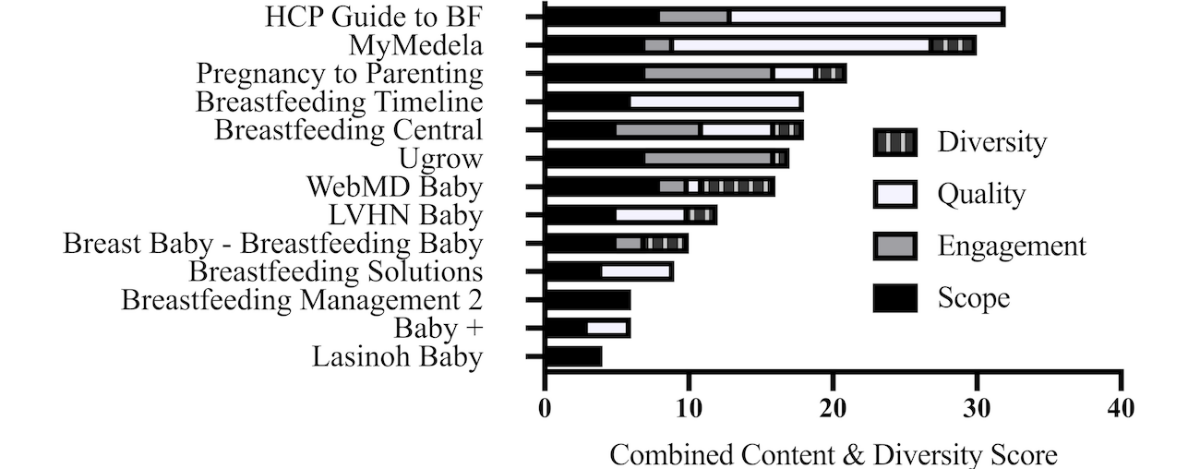
Combined content and diversity scores (n=13). HCP: health care provider; BF: breastfeeding; LVHN: Lehigh Valley Health Network Baby.

Educational content related to milk production (Multimedia Appendix 3) was identified in 13 apps. Seven of these 13 apps also contained features that could assist mothers with promoting, tracking, or interpreting milk production. Since the diversity scoresheet could only be applied to screenshots that contained educational content about milk production, the scores from the content and diversity scoresheets were combined for all analyses. The average combined content and diversity score for all 13 education apps was 15.3 (SD 8.8). To the best of our knowledge, these apps were not created by infant formula companies. The Healthcare Provider’s Guide to Breastfeeding app received the highest combined content and diversity score (32/78), while the Breastfeeding Management 2 app received the lowest combined content and diversity score (4/78). The app with the highest quality of content was the Health Care Provider’s Guide to Breastfeeding, with 79.2% of all the content reviewed containing in-text citations or reference lists to relevant peer-reviewed literature. The apps with the lowest quality of content were Breastfeeding Central and Breastfeeding Management 2; the content reviewed in these apps did not have any references (Figure 2).

**Table 5:** Scores of 18 features related to milk production.

Unique feature (N=18)	Rationale	Score
Baby sound recorder [16,26]	Related to milk production (1 point) AND user engagement *throughout* activity (2 points) AND used ≥1 times/ *week* (2 points)	5
Photos (of baby) [16,26]	Related to milk production (1 point) AND user engagement *throughout* activity (2 points) AND used ≥1 times/ *week* (2 points)	5
Bottle-feeding timer [16,17]	Related to milk production (1 point) AND user engagement *at beginning or end* of activity (1 point) AND used ≥1 times/ *week* (2 points)	4
Breastfeeding timer [16,17]	Related to milk production (1 point) AND user engagement *at beginning or end* of activity (1 point) AND used ≥1 times/ *week* (2 points)	4
Breast-pumping timer [16,17]	Related to milk production (1 point) AND user engagement *at beginning or end* of activity (1 point) AND used ≥1 times/ *week* (2 points)	4
Diaper-change tracker [16,17]	Related to milk production (1 point) AND user engagement *at beginning or end* of activity (1 point) AND used ≥1 times/ *week* (2 points)	4
Sleep pattern [16,17]	Related to milk production (1 point) AND user engagement *at beginning or end* of activity (1 point) AND used ≥1 times/ *week* (2 points)	4
To-do list or reminders [27]	Related to milk production (1 point) AND user engagement *at beginning or end* of activity (1 point) AND used ≥1 times/ *week* (2 points)	4
Baby’s mood [28]	Related to milk production (1 point) AND user engagement *at beginning or end* of activity (1 point) AND used ≥1 times/ *month* (1 point)	3
Allergy [29]	Related to milk production (1 point) AND either user engagement *at beginning or end* of activity (1 point) OR used ≥1 times/ *month* (1 point)	2
Doctor visits [30]	Related to milk production (1 point) AND either user engagement *at beginning or end* of activity (1 point) OR used ≥1 times/ *month* (1 point)	2
Growth [31]	Related to milk production (1 point) AND either user engagement *at beginning or end* of activity (1 point) OR used ≥1 times/ *month* (1 point)	2
Milestones [32]	Related to milk production (1 point) AND either user engagement *at beginning or end* of activity (1 point) OR used ≥1 times/ *month* (1 point)	2
Illness or temperature [33]	Related to milk production (1 point) AND either user engagement *at beginning or end* of activity (1 point) OR used ≥1 times/ *month* (1 point)	2
Baby’s medication tracker [34]	Related to milk production (1 point) AND *minimal to no* user engagement (0 points) AND used <2 times/*month* (0 points)	1
Teeth [34-36]	Related to milk production (1 point) AND *minimal to no* user engagement (0 points) AND used <2 times/*month* (0 points)	1
Record weight/height/date of birth [16,17]	Related to milk production (1 point) AND *minimal to no* user engagement (0 points) AND used <2 times/*month* (0 points)	1
Record singleton/multiples birth [16,17]	Related to milk production (1 point) AND *minimal to no* user engagement (0 points) AND used <2 times/*month* (0 points)	1

A total of 48 photos of the breastfeeding dyad were identified within the screenshots containing educational content related to milk production. Of these, 87.5% (n=42) were of white women and infants. Only 12.5% (n=6) of the photos were of nonwhite women (n=5) or of a nontraditional caretaker—the father (n=1).

The interrater percent agreement between AS and SS on combined content and diversity scoring was 90.23%. The majority of disagreements between the authors was on scoring within the diversity scoresheet. Although diversity represented 8% of the combined content and diversity score, it represented over 20% of the disagreements between AS and SS. The discrepancies were due to disagreements about what aspects of diversity should be quantified (gender, race/ethnicity, and pumping vs direct feeding).

**Figure 3:**
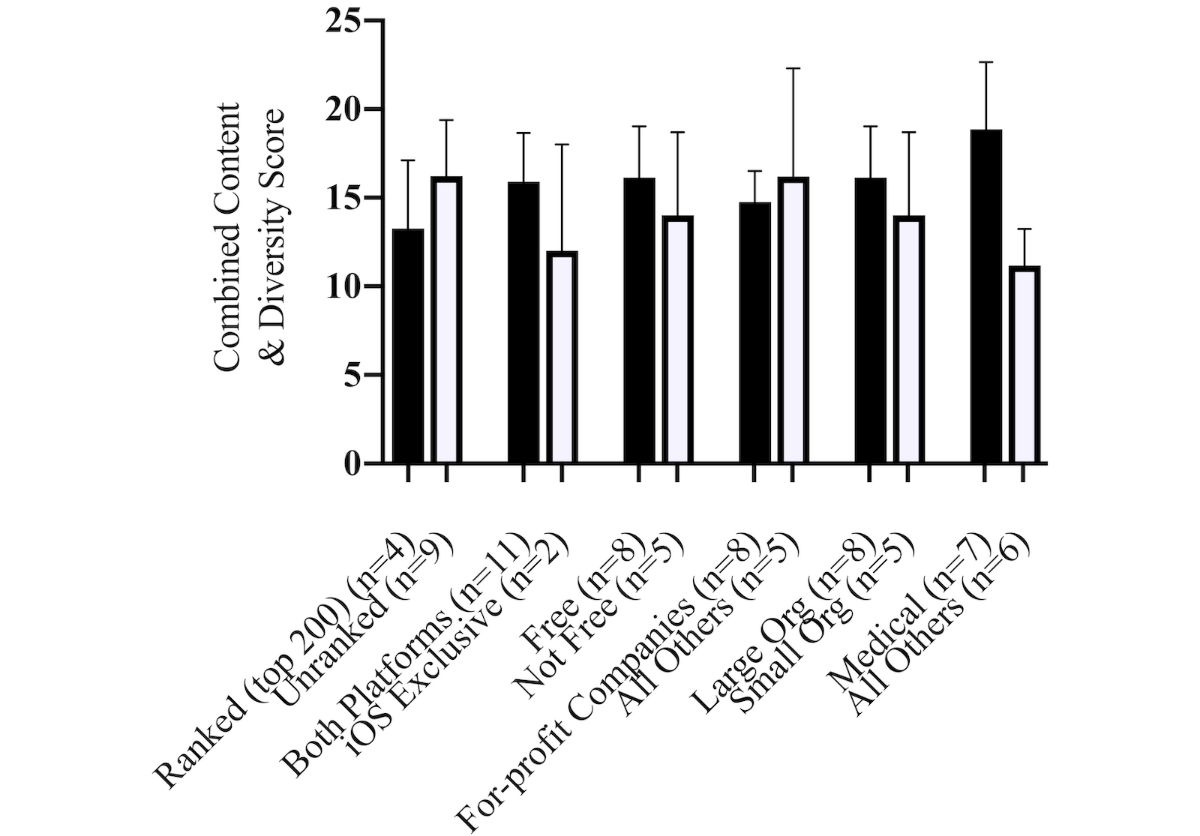
Differences in mean combined content and diversity scores, stratified by app type, category, and organization. iOS: iPhone operating system; Org: organization.

### Characteristics of Apps Containing Educational Content About Milk Production

Among the apps that contained educational content about milk production, there were no differences (Figure 3) in the mean combined content and diversity scores between the apps that were free and not free (*P*=.61), created by for-profit companies and any other organizations (*P*=.77), developed by a small organization and a large organization (*P*=.61), or between apps ranked in the top 200 and unranked apps (*P*=.88). Large organizations (>10 employees) were much more likely to create free apps (*r*=1.0, *P*<.001). There was no correlation between the organization type and business size, platform, or price of the app.

## Discussion

### Principal Findings

In this novel breastfeeding app review, we identified and evaluated features and educational content related to milk production and quantified the diversity of images of breastfeeding experiences within selected screenshots. Although Social Cognitive Theory has been widely used to develop and implement successful health and breastfeeding interventions, to our knowledge, we are the first to use Social Cognitive Theory to inform our evaluation of features, content, and images within smartphone apps [8]. We identified a dearth of high-quality textual and multimedia educational content related to milk production within selected screenshots of breastfeeding apps. Although previous breastfeeding app reviews did not specifically focus on content related to milk production, Taki et al used criteria centered around quality, comprehensibility, suitability, and readability to conclude that educational information within infant-feeding apps was not evidence-based [37]. Using these criteria, they found that 78% of infant-feeding apps (36/46) received a “low-quality” rating because the content was not credible or comprehensive [36]. The average combined content and diversity score among the 13 breastfeeding apps reviewed in our study was 15.3 of 78 points; thus, our findings of low-quality educational content within breastfeeding apps are in agreement with the conclusions of Taki et al about content in infant-feeding apps.

A review of milk supply or milk production educational materials in digital or print media or resources has never been performed. Our results indicate that there is ample room for improvement in the development and delivery of educational content related to milk production within breastfeeding apps. The majority of breastfeeding apps in our review did not reference peer-reviewed scholarly literature. For example, in the Pregnancy and Parenting app, an app by Lamaze International, the user was referred to a website that referenced “The Official Lamaze Guide: Giving Birth with Confidence.” Although most breastfeeding apps broadly covered content within all eight categories, the information provided was superficial and poorly cited. There was a surprising lack of images or videos to help describe educational content related to milk production. As multimedia can be helpful in explaining difficult concepts, like supply-demand physiology or breastfeeding techniques [20,21], we hope that this review will stimulate developers to incorporate engaging breastfeeding educational content into apps.

The features, content, and general characteristics identified in this review can help consumers make informed breastfeeding app purchases by outlining features related to promoting, tracking, interpreting, or teaching about milk production. The top three features (breastfeeding timer, bottle feeding timer, and diaper changes) were included in 85.7% of all reviewed apps. However, only seven apps contained both features and educational content related to milk production. Among these seven apps, four were created by breast pump companies. Without high-quality educational content about milk production within apps containing features that allow for milk production tracking, mothers may interpret the data incorrectly and assume that they are not producing enough milk [6,7,9]. Thus, breastfeeding app creators could use the scoresheets and key findings within this review to develop apps that integrate features to promote breastfeeding self-efficacy with high-quality educational content related to milk production.

In line with the work of Schindler-Ruwisch et al, we found that for-profit companies and large businesses are more likely to create free breastfeeding apps [38]. Schindler-Ruwisch et al completed a content analysis of 53 breastfeeding apps in which they established that the majority of breastfeeding apps were free and developed by for-profit organizations [38]. Their review was limited in scope because the apps were not downloaded and explored; thus, features and educational content were not comprehensively assessed. Moreover, Schindler-Ruwisch et al did not investigate whether for-profit companies developed breastfeeding apps containing features that can assist with tracking milk production or high-quality educational content that aligns with a health behavior theoretical framework [38].

We found that large businesses were more likely to create apps that received a high combined content and diversity score and for-profit companies were more likely to create apps that received a high score on the features scoresheet. Likely due to the small sample size of our dataset, a large business (>10 employees) was not associated with for-profit status. Only one app (Similac; Abbot, IL) in our dataset was created by an infant formula company. Although Similac’s features were evaluated, none of its content was related to milk production. Therefore, it was not included in our educational content review.

To our knowledge, we are the first to report a dearth of breastfeeding imagery diversity within smartphone apps. The lack of diversity in the portrayal of the breastfeeding experience has been reported within print and online media, television, and film [23-25]. Foss found that in television and film, breastfeeding was depicted in a positive light; however, the majority of the breastfeeding characters were educated, older Caucasian women [23]. In British newspapers and television programs, breastfeeding was associated with upper-class and celebrity parents, while formula feeding was a normal, not embarrassing or problematic, infant-feeding alternative [39]. The diversity scoresheet developed for this review is similar to the method that Frerichs et al used for scoring breastfeeding images within popular magazines in the United States [25]. Frerichs et al awarded points to images if they contained pictures of a parent or parents with a baby, whether breastfeeding, bottle feeding, or not feeding [25]. Additionally, they awarded points to images of different races (white, African American, or other). The majority of the people pictured in the images identified by Frerichs et al within popular magazines were white (77.8%), with African Americans in 20.8% of the photos [25]. Frerichs et al concluded that this heterogeneity was representative of the US population, which was only 12.3% African American in 2000 [25]. Of the 241 images reviewed by Frerichs et al, the majority (n=197, 81.4%) were of the mother and her infant and 19% (n=46) were of fathers only [25]. In line with the conclusions of Foss and Frerichs et al, we speculate that it is unlikely that low-income women, minority women, and nontraditional caretakers (grandmothers, fathers, adoptive mothers, etc) would feel empowered to meet their infant-feeding goals using the breastfeeding apps in our review.

### Limitations

The most significant limitation of our approach is the small sample size due to the narrow inclusion criteria and the inability to evaluate Android-exclusive apps. There was significant bias in the data-extraction process, as only one author (SS) identified features related to milk production. Some apps included in the review were based on a freemium business model, meaning that some sections and features of the app were available for free, while other features required a payment to be unlocked. All analyses in this review included features available only in the freemium version; thus, these apps were analyzed within the “free” category. It is unknown how the features and combined content and diversity scores of these apps would have changed if the premium features were unlocked and explored. Apps were divided into two categories: those created by for-profit companies and those created by all other organizations, including nonprofits, individuals, and government. Businesses whose employee information could not be found were assumed to have fewer than 10 employees and stratified into that group for analysis. Thus, our comparisons of different groups may not accurately represent the apps and app developers within that group.

The features, content, and diversity scoresheets were not validated in this study; thus, additional studies will be needed to validate our approach for feature and content evaluation related to milk production within breastfeeding apps. By design, the content scoresheet focused exclusively on assessing the leading cause of breastfeeding cessation—perceived or real low milk production. Thus, it is possible that apps with high-quality content on a different breastfeeding topic were misrepresented as “low quality” due to our targeted content evaluation. Another important limitation of the content scoresheet lies in its inability to encompass how comprehensively or correctly a certain topic was covered. For example, some apps provided dozens of examples of biologic causes of low milk production, while others only mentioned one. In both cases, these apps would receive a point for addressing content within the “Biology or Behavior” category. Incorrect or outdated information was frequently found in apps; however, this was not counted against the developer, as the content scoresheet did not contain a quantitative way of measuring incidence of faulty information provision. The qualifications of the content creators within the apps were not further investigated by the authors. For example, a point was awarded within a category if the author had an advanced degree; however, we did not examine whether that individual’s advanced degree was in a field relevant to the topic about which they were writing. The authors used percent agreement as the measurement of interrater reliability, which does not take into consideration chance agreement.

Finally, a substantial limitation of our approach is that none of scoresheets (features, content, and diversity) incorporated a method of evaluating user interface, user experience, or app design. At times, poor design obstructed our ability to efficiently evaluate content or assess features. We recommend that app developers conduct sufficient user testing to ensure easy identification of features and content, progress from one screen to the next in a logical manner, and minimalistic and simple design to reduce clutter on each screen.

### Conclusions

To our knowledge, we are the first to identify and evaluate, using scoresheets informed by Social Cognitive Theory, the features and content related to milk production in 41 breastfeeding apps. We identified a dearth of high-quality, engaging, educational content related to milk production. The majority of the breastfeeding imagery within the screenshots containing educational content about milk production depicted white women; thus, it is likely that parents, especially those from minority or low-income groups, have limited options to choose from when selecting an app to reach their breastfeeding goals. For-profit companies and large organizations were most likely to create free apps that received high scores on the combined content and diversity or features scoresheet, respectively. The findings in this review will be useful for health care providers and parents when recommending or selecting breastfeeding apps, respectively, and for app creators when developing content and features to increase breastfeeding self-efficacy.

## Appendix

Multimedia Appendix 1 Breastfeeding app and app creator/organization description.

Multimedia Appendix 2 Comprehensive list of features identified within breastfeeding apps.

Multimedia Appendix 3 Eight content categories related to milk production.

Multimedia Appendix 4 Breastfeeding app–selection process.

Multimedia Appendix 5 Reasons for excluding select breastfeeding apps.

Multimedia Appendix 6 General characteristics of breastfeeding apps and app developers.

Multimedia Appendix 7 Final breastfeeding app dataset (N=41).

## Abbreviations

SCT: Social Cognitive Theory
