# Supplementary material for: Features and Educational Content Related to Milk Production in Breastfeeding Apps: Content Analysis Informed by Social Cognitive Theory
Source: JMIR Pediatr Parent. 2019 May 1;2(1):e12364. doi: 10.2196/12364 (PMC6715395; doi:10.2196/12364)
Supplement: Multimedia Appendix 1 [file pediatrics_v2i1e12364_app1.pdf]

S1: Breastfeeding app and app creator/organization description

1. App category (medical, health and fitness, lifestyle, or productivity)
2. App creator/organization
3. App ranking in App Store (between 1-200 or not ranked)
4. Company website
5. Date app was created
6. Date of last update
7. Description of app logo (color, image, text)
8. Description of developer/organization
9. Estimated number of Android downloads
10. Estimated number of employees
11. Estimated number of iOS downloads
12. Estimated number of total downloads
13. Estimated number of updates
14. Location of creator/organization
15. Motivation for creating app
16. Name of developer/organization
17. Name of app
18. Number of Android ratings
19. Number of iOS ratings
20. Number of total ratings
21. Organization's/creator's email
22. Organization's/creator's phone number
23. Other products created by organization (other apps, services, devices, etc.)
24. Platform (iOS or Both iOS and Android)
25. Storage required (megabytes)
26. Summary rating (1-5 stars)
27. Type of Organization (Government, Individual, Non-profit, Private Corporation (for-profit), Public Corporation (for-profit))
