# Supplementary material for: Features and Educational Content Related to Milk Production in Breastfeeding Apps: Content Analysis Informed by Social Cognitive Theory
Source: JMIR Pediatr Parent. 2019 May 1;2(1):e12364. doi: 10.2196/12364 (PMC6715395; doi:10.2196/12364)
Supplement: Multimedia Appendix 2 [file pediatrics_v2i1e12364_app2.pdf]

S2: Comprehensive list of features identified within breastfeeding apps

1. Allergy*
2. Baby activity tracker
3. Baby bath tracker
4. Baby sound recorder*
5. Baby vaccination tracker
6. Baby's mood*
7. Baby’s medication tracker*
8. Blood type
9. Bluetooth connectivity
10. Bookmark/favorites
11. Bottle feeding timer*
12. Breast pumping timer*
13. Breastfeeding timer*
14. Calendar access
15. Data backup
16. Data export
17. Data sync
18. Diaper change tracker*
19. Diary
20. Disclaimer (Eg. health and data privacy, data use, medical advice/education)
21. Doctor Visits*
22. Education (of child) tracker
23. Email required for login
24. Formula tracker
25. Forums/chats
26. Growth*
27. Illness or temperature*
28. Link that outsources information to a website within the app (without using the default browser)
29. Location services
30. Milestones*
31. Option to customize background
32. Passcode option
33. Photo everyday
34. Photos (of baby)*
35. Presence of advertisements
36. Presence of in-app purchases
37. Projection of potential milk consumption based on past milk production data input by user
38. Push notifications
39. Quick response (QR) code compatibility
40. Record singleton/multiples birth*
41. Record weight/height/date of birth*
42. Required internet connection
43. Required sign up to third party to use app
44. Sleep pattern*

45. Teeth*
46. Tips/tricks/news
47. To do list or reminders*
48. Tummy time
49. Tutorial/demo of app
50. User data graph generator
51. Video (within the app)
52. Video consultant
53. Weather detection
54. Widget capability

Features with an asterisk (*) were selected by the authors as “milk production-related” as shown in Table 5.
