# Supplementary material for: Features and Educational Content Related to Milk Production in Breastfeeding Apps: Content Analysis Informed by Social Cognitive Theory
Source: JMIR Pediatr Parent. 2019 May 1;2(1):e12364. doi: 10.2196/12364 (PMC6715395; doi:10.2196/12364)
Supplement: Multimedia Appendix 3 [file pediatrics_v2i1e12364_app3.pdf]

S3: Eight content categories related to milk production

**Hospital practices that promote breastfeeding initiation**
Discussion of interventions, education, or activities that promote breastfeeding prenatally or in the first zero to five days postpartum (eg. anticipatory counseling, lactation consultant visit, and/or breastfeeding class)
Discussion of the importance of early breastfeeding initiation, on demand, frequent breastfeeding (8-12 times/day) in hospital within first hour/first 24 hours
No separation of mother-baby after delivery
No oral intake of fluids, unless medically necessary, other than breast milk
Discussion of the importance of skin-to-skin contact
**Reasons for delay in lactogenesis II**
Maternal systemic conditions (eg. insulin resistance), stress, separation of mother-baby, complications of labor/delivery, maternal surgical or medical history (eg. radiation of chest), maternal anatomy leading to decreased production or poor latch, and/or maternal medications (eg. drowsiness, breastfeeding contraindication)
Infant anatomy leading to poor suck, infant illness leading to separation, and/or prematurity (poor suck/separation)
**Normal milk production timeline, volume, and measurements**
Lactogenesis I in second trimester, lactogenesis II around 72 hours postpartum
Average milk production in first week (~100 mLs day one to ~500 mLs day five) and between months one and six postpartum (~780 mls/day)
Weighted feeds, pump volumes, breast storage capacity as estimates of milk production
**Supply and demand physiology**
Supply & demand mentioned (various synonyms of supply and demand acceptable)
Discussion of hormonal influence on milk production (prolactin, oxytocin, cortisol, insulin)
**Maternal or infant nutritional requirements**
Discussion of increased caloric requirements for mother (~300 additional calories) and increased or modified macro-micro-nutrient requirements
Discussion of milk composition changes as related to infant nutritional needs for optimal growth
Discussion of average infant intake (no increase between one month and six months postpartum: 24-30 oz./day)
**Breastfeeding techniques that support or interfere with milk production**
Discussion of optimal or suboptimal techniques: pumping, on demand feeding, infant cues, latch assessment, positioning
**Biological/physiological/behavioral reasons for perceived or real low milk production (before six months postpartum)**
Maternal systemic conditions (eg. insulin resistance), stress, separation of mother-baby, complications of labor/delivery, maternal surgical or medical history (eg. radiation of chest), maternal anatomy leading to decreased production or poor latch, and/or medications that decrease production

Infant anatomy leading to ineffective suck, infant illness leading to separation, prematurity (poor suck/separation), medications that decrease infant alertness, neurological (eg. Downs syndrome), growth spurt (eg. cluster feeding causing mother to think milk supply is not enough), early complementary food introduction, and/or behavioral (eg. breast aversion, bottle preference)
**Foods/Medications/Supplements that have the potential to increase milk production**
Discussion of foods, medications, and/or herbs that have the potential to increase milk production
