# Supplementary material for: Features and Educational Content Related to Milk Production in Breastfeeding Apps: Content Analysis Informed by Social Cognitive Theory
Source: JMIR Pediatr Parent. 2019 May 1;2(1):e12364. doi: 10.2196/12364 (PMC6715395; doi:10.2196/12364)
Supplement: Multimedia Appendix 4 [file pediatrics_v2i1e12364_app4.pdf]

**S4.** Breastfeeding app selection process

| Action taken | # of apps | # of apps excluded | Reason for exclusion |
|---|---|---|---|
| Searched "breastfeeding" in App Store | 105 | 34 | Summary rating unavailable: 32<br>Not available in the United States: 2 |
| Qualifying Screen | 71 | 18 | Free trials: 9<br>Technical glitches: 7<br>Not in English: 2 |
| First and Second Review | 53 | 6 | Deleted: 4<br>Not available in the United States: 2 |
| Final Assessment | 47 | 6 | Lack of content related to milk production: 6 |
| Included in Data Set | 41 | 0 | N/A |
