# Supplementary material for: Features and Educational Content Related to Milk Production in Breastfeeding Apps: Content Analysis Informed by Social Cognitive Theory
Source: JMIR Pediatr Parent. 2019 May 1;2(1):e12364. doi: 10.2196/12364 (PMC6715395; doi:10.2196/12364)
Supplement: Multimedia Appendix 5 [file pediatrics_v2i1e12364_app5.pdf]

S5: Noteworthy educational app exclusions

Name of App	Description of App	Rationale for Exclusion
Infant Risk Center Healthcare Mobile	Medications and herbal supplements manager	Did not provide information about milk production beyond medications and supplements.
LactMed		
Mom Community Chat	Forum for mothers to discuss their experiences	Educational content was not purposefully included in the app by the app developers
Moms Pump Here	Nursing/lactation room locator	Did not have any educational content directly related to milk production
Mamava		
LatchME	Telemedicine app that has the potential to connect an end user to relevant resources	No written content related to milk production.
