# Supplementary material for: Features and Educational Content Related to Milk Production in Breastfeeding Apps: Content Analysis Informed by Social Cognitive Theory
Source: JMIR Pediatr Parent. 2019 May 1;2(1):e12364. doi: 10.2196/12364 (PMC6715395; doi:10.2196/12364)
Supplement: Multimedia Appendix 6 [file pediatrics_v2i1e12364_app6.pdf]

S6: General characteristics of breastfeeding apps and app developers

General characteristics	N = 41 (%)
Types of apps	
Apps that contain features that assist mothers with tracking or interpreting milk production	28 (68.4)
Apps that only have educational content about milk production.	6 (14.6)
Combination Apps: Apps that contain features that assist mothers with tracking or interpreting milk production <u>and</u> have educational content about milk production.	7 (17)
Free and "not free" apps	
Free Apps: free to download and use	30 (73.2)
"Not Free" Apps: require payment to download and use	11 (26.8)
Average price	$3.44
Price range	$0.99-$4.99
Platform	
iOS only: Available only in the App Store	21 (51.2)
iOS and Android: Available on App Store and Google Play Store	20 (48.8)
App Developer or Organization	
Government	1 (2.5)
Individual	11 (26.8)
Non-profit	1 (2.5)
Private Corporation (for-profit)	20 (48.8)
Public Corporation (for-profit)	8 (19.5)
Business Size: The number of employees that work for the organization that developed the app.	

Unknown	5 (12.2)
<10	25 (60.9)
10 < x < 50	4 (9.7)
50 < x < 250	1 (2.5)
250 < x < 1000	1 (2.5)
1000+	5 (12.2)
Ranking in the top 200 on the App Store: Breastfeeding apps in our dataset that have overall positive user reviews are ranked in the top 200 within their respective category (medical, health and fitness, lifestyle, education, productivity) in the App Store.	
Breastfeeding apps not ranked in their category	30 (73.2%)
Breastfeeding apps ranked in the top 200 in their category	11 (26.8%)
App Category in App Store: Using the App Store, app developers can assign a primary category that best describes an app’s main purpose. The primary category influences the filters placed on search results and determines an app's placement within the App Store.	
Medical: Apps that are focused on medical education, information management, or health reference for patients or healthcare professionals.	23 (56.1%)
Health and Fitness: Apps related to healthy living, included stress management, fitness, and recreational activities.	14 (34.1%)
Lifestyle: Apps relating to a general-interest subject matter or service.	3 (7.3%)
Productivity: Apps that make a specific process or task more organized or efficient.	1 (2.5%)
