# Supplementary material for: Features and Educational Content Related to Milk Production in Breastfeeding Apps: Content Analysis Informed by Social Cognitive Theory
Source: JMIR Pediatr Parent. 2019 May 1;2(1):e12364. doi: 10.2196/12364 (PMC6715395; doi:10.2196/12364)
Supplement: Multimedia Appendix 7 [file pediatrics_v2i1e12364_app7.pdf]

S7: Final breastfeeding app data set (N = 41)

Name of App	Features Score	Combined Content and Diversity Score
**Awesome Baby Tracker**	41	
**Baby +**	47	21
**Baby Daily Activity Tracker**	41	
**Baby Feed Timer - BF Baby Tracker**	33	
**Baby Feeding Tracker - Hello Baby**	22	
**Baby Log - Record Your Newborn's…**	20	
**Baby Tracker Pro Feed Timer**	34	
**Baby's Day by Winnie**	21	
**Babycare Tracker - Baby Activities and**	40	
**BabyNursing**	37	
**BabyTime Baby Feeding Timer - BF**	20	
**BabyTracker**	36	
**BF tracker, breast pumping, feeding app**	32	
**Breast Baby - Breastfeeding Baby**	-	18
**Breastfeeding**	4	
**Breastfeeding Central**	-	6
**BreastFeeding Friend**	12	
**Breastfeeding Management 2**	-	4
**Breastfeeding Solutions**	-	12
**Breastfeeding Timeline**	-	30

**Eat Sleep**	25	
**Feed Baby**	37	
**Glow Baby**	41	
**Hatch Baby**	32	
**Lasinoh Baby**	22	16
**LVHN Baby**	19	17
**MyMedela**	32	18
**New Baby Feeding Log**	16	
**Newborn Baby Tracker: Breastfeeding**	20	
**Nursing Monitor**	28	
**Nursing Notebook**	33	
**Parentlove: Newborn Tracker**	40	
**Pregnancy to Parenting**	19	9
**Serene Breastfeeding Timer**	4	
**Similac Baby Journal**	30	
**Sprout Baby + Feeding**	35	
**The Health Care Provider's Guide to Breastfeeding**	-	32
**Time for Baby - Breastfeeding and**	16	
**uGrow Baby Development Tracker**	35	10
**WebMD Baby**	29	6
**Which Boob?**	4	
